# The prevalence of moderate to severe radiographic sacroiliitis and the correlation with health status in elderly Swedish men – The MrOS study

**DOI:** 10.1186/1471-2474-14-352

**Published:** 2013-12-13

**Authors:** Sofia Exarchou, Inga Redlund-Johnell, Magnus Karlsson, Dan Mellström, Claes Ohlsson, Carl Turesson, Lars Erik Kristensen, Lennart Jacobsson

**Affiliations:** 1 Department of Clinical Sciences, Lund University, Malmö, Sweden; 2 Department of Radiology, Skåne University Hospital, Lund, Sweden; 3 Department of Orthopedics and Molecular Osteoporosis Research Unit, Skåne University Hospital, Malmö, Sweden; 4 Center for Bone and Arthritis Research at the Sahlgrenska Academy, Sahlgrenska University Hospital, Gothenburg, Sweden; 5 The Sahlgrenska Academy, University of Gothenburg, Rheumatology anf Inflammation Research, Institute of Medicine, Gothenburg, Sweden; 6 Rheumatological Department, University Hospital of Skåne, Inga Marie Nilssons gata 32, 205 02 Malmö, Sweden

## Abstract

**Background:**

Ankylosing Spondylitis (AS) is a chronic inflammatory disease with onset in young adults, but little is known about the prevalence in older age groups. Furthermore, there is very limited information of health status of elderly patients with AS. Our objective was to estimate the prevalence of moderate to severe radiographic sacroiliitis in elderly men and its impact on health.

**Methods:**

A cross-sectional, population-based survey, that included 1005 men aged 69-81 years old, with the primary aim to study risk factors for osteoporosis (MrOS), was used. X-rays of the pelvis and spine were done for the whole population and then examined by two readers. The prevalences of grade 3-4 sacroiliitis, syndesmophytes and spondylophytes were ascertained. Using a self-administered questionnaire, information was obtained on physical activity (PASE), functional status (IADL items), health related quality of life - QoL (SF-12) and back pain (pain question, Quebec Pain Disability Scale items).

**Results:**

Fourteen cases with grade 3-4 sacroiliitis were identified, corresponding to a prevalence of 1.4% (95%CI: 0.7-2.4). Eight of the patients with sacroiliitis had both AS-typical and degenerative changes in the spine, 4 had only degenerative changes and 2 had only AS-related changes. There were no statistically significant differences between those with and without radiographic sacroiliitis regarding demographics, anthropometric measures, smoking status or health status, reflected by measures on physical activity, functional status, health related QoL and back pain.

**Conclusions:**

The prevalence of moderate to severe radiographic sacroiliitis was estimated to be 1.4% among elderly men in Sweden. Self-reported health was only slightly different in those with sacroiliitis, suggesting that the relative impact of AS is modest in this age group.

## Background

Ankylosing Spondylitis (AS) is a chronic inflammatory disorder primarily involving the sacroiliac (SI) joints and the axial skeleton. It is the prototype disease of a large disease family named Spondyloarthrities (SpA) [1]. The first spondyloarthritis symptom occur in about 80% of patients before the age of 30 and less than 5% of patients present with the disease after the age of 45 years [2]. The male: female ratio is about 2:1 [2].

The hallmark for the diagnosis of AS, according to the modified New York (mNY) criteria has been the detection of sacroiliitis by plain radiographs [3]. Sacroiliitis is graded as 0-4 (normal, suspicious changes, minimal abnormality, unequivocal abnormality, total ankylosis). Sacroiliitis at least grad 2 bilaterally or grade 3 unilaterally, and simultaneously the presence of either chronic inflammatory back pain, restricted spinal mobility or chest expansion is needed for the diagnosis to be made according to those criteria [3].

The recognition of structural changes in sacroiliac joints is a challenge and requires expertise and experience, because of the complex anatomy of the sacroiliac joints, the two-dimensional capacity of conventional radiography and the addition of degenerative changes with increasing age. In line with this, inter- and intra-observer variations have been reported to be large, especially with regard to grades 1 and 2, while the concordance rates were higher if the SI-joints were definitely normal (grade 0) or definitely abnormal (grade 4) [4].

Different study designs have been used in the available studies on the prevalence of AS. Some studies screened HLA-B27-positive and negative blood donors [5-8] or the patients’ relatives [9]. Other studies used hospital records [10,11], registry data [12-14] or population surveys [15-22]. Most of the population-based studies proceed from identifying the subjects with joint or back pain to the identification of people with radiographically verified AS. Considering the fluctuating nature of the disease associated symptoms and the relatively long lag time between symptoms onset and diagnosis, underestimation of the real prevalence is a risk, when using this approach.

The prevalence of AS is strongly dependent on the prevalence of HLA-B27 in a given population. Prevalence estimates of 0.03–1.8% have been described for AS, with most of these data coming from Europe [5,10-21,23]. Current estimates for the prevalence of AS in the United States (US) range between 0.2% - 0.5% [24].

There are only a few population studies on the prevalence of radiographic sacroiliitis. A high prevalence was found in the adult males of the Haida Indians and other Native American tribes [25]. The results from the first National Health and Nutrition Examination Survey (NHANES I), a population based study performed between 1970 and 1975 in the US, indicated that the prevalence of severe to moderate radiographic sacroiliitis in men was 7.3/1000 for ages 25–74 years and in women 3.0/1000 for ages 50–74 years [26,27].

Prevalence studies that have been based on a sequential approach identifying subjects having disease by the presence of symptoms with later confirmation with radiographs, have suggested decreasing prevalence over the age of 65 [17,21]. Similar results have been found in studies based on hospital records [11] and registries [13]. Whether such a decline is due to decreasing symptoms of Spondyloarthritis with age or an overshadowing increase in degenerative back problems and other comorbidities is not clear, since there is very limited information of health status of elderly patients with AS or other types of SpA.

The aim of the present study was to investigate the prevalence of moderate to severe (grade 3 and 4) radiographic sacroiliitis among men aged 69-81 years and in addition to compare the health status in this group with that of men of similar age from the same population survey.

## Methods

### Study population

The population in the present study is collected from the MrOS (Mister Osteoporosis) Sweden cohort [28]. MrOS Sweden is part of an international, multi-center, prospective, longitudinal, observational study with primary aim to identify risk factors for osteoporosis and fractures (vertebral and non-vertebral) in old men, and was additionally designed to determine how fractures affect quality of life in men [29]. The study was conducted in three Swedish cities, Uppsala (n = 999), Malmo (n = 1005) and Gothenburg (n = 1010) and was performed in accordance with the Declaration of Helsinki. The ethics committee of Lund, Sweden, approved the study and all participants gave written informed consent [28].

The MrOS Malmo Study enrolled 1005 men, aged 69–81 years, between October 2001 and December 2004. Invitations were sent by mail to 2065 men who were living in Malmo and who were randomly selected from the national population registry resulting in an attendance rate of 48.7% [30].

Based on the inclusion criteria for the MrOS study, the participants should be able to walk without the assistance of another person, had not undergone bilateral hip replacement operation, be able to provide self-reported data, be able to understand and sign an informed consent and not suffer from a medical condition that (in the judgment of the investigator) would result in imminent death [29].

### Assessment of sacroiliitis and spinal changes

Spinal radiographs and radiographs of the pelvis including both hip-joints were obtained for all the participants. For the spinal images, the participants were placed on their left side in the lateral position and the images included the vertebra from T2 to S1. An experienced radiologist (IRJ) screened and scored the anteroposterior pelvic radiographs for sacroiliitis. The cut-off for a positive x-ray was unilateral sacroiliitis grade 3, as defined by mNY criteria [3], because of the expected poor inter- and intra-observer concordance in grades 1 and 2 [4]. An additional reader (SE) reviewed the positive x-rays blinded for the primary scoring. There were no discordances between the two readers. The images where sacroiliitis was present were then reviewed by both readers for the presence of AS-related changes (syndesmophytes and bridges) and degenerative changes (spondylophytes and bridges). In consistence with previous proposals, the AS-related changes (syndesmophytes and bridges) were defined by a horizontal growth angle of <45° to the anterior vertebral side [31,32]. The spinal discs were only qualitively evaluated since to our knowledge, there is not a widely accepted quantitative definition for radiographic disc degeneration.

### Self-reported measures

Information on demographics, anthropometrics, lifestyle, physical activity, functional status, health-related quality of life and back-health were obtained from a self-administered questionnaire.

Physical Activity was assessed by the Physical Activity Scale for the Elderly (PASE) questionnaire which consists of self-reported occupational, household and leisure activities items commonly engaged in by elderly persons [33]. It is a valid method to classify healthy elderly people into levels of physical activity [34]. PASE scores may range from 0 to 400 or more. Higher scores indicate a higher level of physical activity. The instructions for use and scoring given in the PASE Administration and Scoring Manual were followed [35].

Functional status was assessed with the use of two questions (being able to 1. walk two to three blocks outside on level ground and 2. climb 10 steps unassisted and without using special aids) from the instrumental activities of daily living (IADL) questionnaire [36].

The Short Form Health Survey (SF-12) was used to measure perceived health and health-related quality of life (QoL) that describes the degree of general physical health status and mental health distress. Separate summary scores are obtained for each of the physical (physical component score, PCS12) and mental (mental component score, MCS12) domains. Both PCS12 and MCS12 can range between 0-100 (mean 50 ± 10). Higher scores indicate higher levels of health [37].

History of any back pain in the last 12 months was assessed with a direct question (yes/no). Secondly, five items from the Quebec Pain Disability Scale were used, in which participants reported on difficulties with bending down to pick up lightweight objects, putting socks on either foot, getting in or out of the front seat of a car, standing for 2 hours, or sitting for 30 minutes because of back pain.

### Analysis of non-participants

In order to address a potential selection bias and assess the generalizability of the results of this study to the whole male population of this age group, we obtained complementary permission from the local ethics committee, to conduct an analysis of non-participants (n = 1060). Diagnoses codes (ICD10) from out-patient visits to physicians and hospitalization for cardiovascular disease (CVD), stroke, diabetes, chronic obstructive pulmonary disease (COPD), AS/SpA and rheumatoid arthritis (RA) were identified for the whole population (n = 2065) for the year of invitation to the study using the population based Regional Outpatient and Inpatient Health Care Registry [38] and a comparison between those enrolled in the study and those who did not participate was carried out.

### Statistical methods

All statistical analysis was conducted using SPSS software. In descriptive analyses, distributions of baseline characteristics, physical activity score (PASE), functional status (IADL), Quality of Life (SF-12) and pain characteristics (any pain, Quebec items) between men with and without evident radiographic sacroiliitis, were estimated using Mann Whitney U non-parametric test for continuous variables and Chi- 2 or Fisher’s exact test for categorical variables. P-values < 0.05 were considered significant.

## Results

### Prevalence

Fourteen cases with moderate to severe (grade 3-4) radiographic sacroiliitis were identified corresponding to a prevalence of 1.4% (95% confidence interval - CI: 0.7-2.4). There were 7 with sacroiliitis grade 3 bilaterally, 4 with grade 4 bilaterally, 1 with grade 3 involvement at one SI-joint and grade 4 at the other and only 1 with unilateral grade 3 involvement (Table 1). The concordance between the two readers was 100%.

**Table 1:** Radiographic sacroiliitis (given as grade 0-4) and spinal radiographic changes given as the number of lesions in the 14 men with moderate to severe radiographic sacroiliitis respectively

**Patient nr**	**Sacroiliitis grade**	**Syndesmophytes**	**Bridges-AS**	**Spondylophytes**	**Bridges-degenerative**
1	Grade 3 bilaterally	0	1	2	2
2^#^	Grade 3 bilaterally	1	1	4	7
3	Grade 3 bilaterally	0	4	0	0
4	Grade 4 bilaterally	10	8	2	1
5	Grade 4 bilaterally	0	14	2	0
6	Grade 2 R /Grade 3 L	5	5	8	0
7	Grade 3 bilaterally	0	9	10	0
8	Grade 3 bilaterally	0	0	8	1
9	Grade 4 bilaterally	4	5	1	0
10	Grade 3 bilaterally	0	0	8	0
11^#^	Grade 4 R /Grade 3 L	0	0	17	6
12	Grade 3 bilaterally	0	0	7	4
13	Grade 4 bilaterally	1	8	0	0
14	Grade 4 bilaterally	0	7	11	1

*The number of AS-related changes (syndesmophytes and bridges) and degenerative changes (spondylophytes and bridges) in the spinal radiographs in those with radiographic sacroiliitis, as defined by the 45° rule. R: Right, L: Left.

#Cases with spinal changes resembling DISH (a. flowing ossification extending over 4 contiguous vertebrae, b. relative preservation of intervertebral disc height in relation to age).

All cases with sacroiliitis had either AS-related changes, such as syndesmophytes and bridges or degenerative changes in the spine radiographs. The majority (8/14) had both types of changes, 4 only degenerative changes and 2 only AS-related changes (Table 1).

Among those, two had spinal findings resembling Diffuse Idiopathic Skeletal Hyperostosis (DISH) (Table 1). Their SI-joints demonstrated sacroiliitis grade 3 or 4. In the absence of CT images we were unable to say if they had a real ankylosis or only ossification of the anterior surface of the joint and consequently fulfilling the DISH criteria [39], therefore these cases were included in the final results.

### Demographic information

At baseline there were no significant differences between men without and those with radiographic sacroiliitis regarding the age and anthropometric measures such as height, weight and body mass index (BMI) nor regarding ethnicity or smoking status (Table 2).

**Table 2:** Demographic characteristics in those with and without evident radiographic sacroiliitis respectively

	**Moderate to severe sacroiliitis (grade 3 or 4)**			
**Characteristics**	**No (N = 991) Median (IQR)**	**Yes (N = 14) Median (IQR)**	**p value**	**Cases with missing information**
**Age (yrs.)**	75.5 (72.8-78.4)	76.3 (73.4-78.0)	0.11	0
**Height (cm)**	174.2 (169.4-178.5)	176.9 (174.0-180.0)	0.10	0
**Weight (kg)**	79.5 (72.4-87.7)	80.3 (76.3-89.8)	0.99	0
**BMI (kg/m**^**2**^**)**	26.3 (24.2-28.6)	25.4 (23.9-28.8)	0.59	0
	**No (n, %)**	**Yes (n, %)**		
**Ethnicity**				
**- Swedish**	927 (93.5)	11 (78.6)	0.06	0
**- Other**	64 (6.5)	3 (21.4)		
**Smoking**				
**- Never**	306 (30.9)	4 (28.6)		
**- Ever**	683 (69.1)	10 (71.4)	1.00**	2/0
**- Current**	108 (15.5)	1 (10)	1.00**	292/4

*IQR: Interquartile range **For ever/never smoking and current/never smoking in those with and without radiographic sacroiliitis. Cases with missing information in the two groups (with and without sacroiliitis).

### Health status

There were no statistically significant differences between the two groups with regard to physical activity, health related quality of life and back pain, although those with radiographic sacroiliitis tended to have a higher frequency of back pain, especially when sitting for more than 30 minutes (Table 3). The majority of individuals with sacroiliitis did not report major difficulties related to back problems (Table 3).

**Table 3:** Health status in those with and without evident radiographic sacroiliitis respectively

	**Moderate to severe sacroiliitis (grade 3 or 4)**			
	**No (N = 991) Median (IQR)**	**Yes (N = 14) Median (IQR)**	**p value**	**Cases with missing information**
**Physical activity**				
PASE score	113.5 (75.7-157.2)	131.7 (52.1-171.8)	0.40	146/1
**Health related quality of Life**				
SF-12				
- PCS12	50.7 (42.0-55.1)	46.1 (35.9-55.9)	0.56	77/2
- MCS12	57.8 (53.0-60.7)	56.1 (48.4-59.8)	0.59	
	**No (n, %)**	**Yes (n, %)**		
**Functional status**				
**IADL1:** Difficulty walking 2-3 blocks without using special aids	122 (12.6)	3 (21.4)	0.41	20/0
**IADL2:** Difficulty climbing up to 10 steps without resting	92 (9.5)	2 (15.4)	0.36	22/1
**History of back pain**				
Any back pain	550 (55.7)	7 (50.0)	0.79	4/0
**Quebec 1:** Difficulty bending down to pick up things because of back pain	243 (24.9)	6 (42.9)	0.13	14/0
**Quebec 2:** Difficulty putting socks on either foot because of back pain	239 (24.2)	5 (35.7)	0.35	5/0
**Quebec 3:** Difficulty getting in or out of the front seat of a car because of back pain	229 (23.2)	5 (35.7)	0.34	5/0
**Quebec 4:** Difficulty standing or walking for 2 hours because of back pain	319 (40.1)	5 (41.7)	1.00	196/2
**Quebec 5:** Difficulty sitting in a chair for 30 minutes because of back pain	103 (10.4)	4 (28.6)	0.05	5/0

### Non-participants

A higher frequency for visits among non-participants to the survey vs. participants with regard to registered ICD10 codes for stroke, diabetes and COPD, and a tendency towards higher rates of CVD was found. However, there were no apparent differences between the two groups with regard to the frequency of visits due to RA or AS/SpA. Only one subject among the non-participants had a visit with an ICD10 code of AS/SpA during the invitation year (Table 4).

**Table 4:** Morbidities diagnosed during the invitation year in participants and non-participants in the MrOS study

	**Non participants (n = 1060)**	**Participants (n = 1005)**	**P value**
**CVD**	116 (10.9%)	85 (8.5%)	0.06
**Stroke**	59 (5.6%)	28 (2.8%)	<0.01
**Diabetes**	86 (8.1%)	44 (4.4%)	<0.001
**COPD**	51 (4.8%)	16 (1.6%)	<0.0001
**AS**	1 (0.1%)	0 (0.0%)	1.00
**RA**	4 (0.4%)	4 (0.4%)	1.00

## Discussion and conclusion

In the present study the prevalence of moderate to severe radiographic sacroiliitis was estimated to be 1.4% among elderly men in Sweden. The cases with radiographic sacroiliitis had similar health status or only a modest deterioration, compared to controls.

In the NHANES I study [26,27], the prevalence of moderate to severe radiographic sacroiliitis was estimated to be 6.0/1000 in men 65-75 years old. Our results indicate a higher prevalence, although the age group is not exactly identical. According to the authors, there may be underestimates due to conservative scoring in the NHANES I study, and the overall grade for sacroiliitis was averaged between bilateral sites, which may have diluted the scores if there was only unilateral changes [26]. Moreover, different grading scales of sacroiliitis were used in our study [3] and the NHANES I study [27], further limiting direct comparability of the results.

The present study did not demonstrate any major, statistically significant, differences in self-perceived health status between those with and without radiographic sacroiliitis, although the small number of cases limits statistical power for formal testing. The finding that the majority of those with sacroiliitis did not report major impairment may have several explanations, including that there is only moderate correlation between syndesmophytes on one hand and function and stiffness on the other [40,41] and that the presence of isolated sacroiliitis in itself may not be clearly associated with function [42]. Furthermore it has also been demonstrated that degenerative changes, e.g. Diffuse Idiopathic Skeletal Hyperostosis (DISH) are not associated with reduced physical activity and health-related QoL or higher level of back pain [43]. In addition, it may be as suggested by others that the proportion of male patients with AS who report severe pain decreases with increasing disease duration [44], possibly due to a decreasing component of active spinal inflammation with increasing age.

Strengths of our study include the population based approach, the standardized radiographic examination of the whole study population and the registry based evaluation of diagnoses in participants as well as non-participants. We specifically addressed health status among older men, where data in the literature are sparse.

Potential limitations include bias due to the relatively large proportion of non-participants. This led us to perform a secondary registry based evaluation of non-participants. As expected, our analysis revealed higher proportions with a health care visit with ICD-codes for CDV, Stroke, Diabetes and COPD among non-participants. However, there were no differences between the groups with regard to RA or AS/SpA, supporting the validity of our prevalence estimates.

Another limitation may be that we did not evaluate the prevalence of grade 2 sacroiliitis or clinical manifestations, which would have enabled definition of AS according to the modified New York Criteria. As a result the prevalence rate estimated in this study is not directly comparable to those of previous studies on prevalence of AS/SpA. Thirdly, the low number of cases with sacroiliitis hampers the possibilities to detect small group differences.

To sum up, we estimated the prevalence of moderate to severe (grade 3-4) radiographic sacroiliitis to 1.4% in a population of elderly men in Sweden. Self-reported health was only slightly different in those with sacroiliitis, suggesting that the relative impact of radiographic sacroiliitis is modest in this age group.

## Abbreviations

MrOS: Mister osteoporosis; PASE: Physical activity scale for the elderly; IADL: Instrumental activities of daily living; SF-12: Short form health survey; PCS: Physical component score; MCS: Mental component score; AS: Ankylosing spondylitis; SpA: Spondyloarthritis; mNY: Modified New York; NHANES: National Health and Nutrition Examination Survey; RA: Rheumatoid arthritis; DISH: Diffuse idiopathic skeletal hyperostosis.

## Competing interests

The authors declare that they have no competing interests.

## Authors’ contributions

SE, LJ, CT, and LEK participated in the study design and in writing the manuscript. MK, DM, CO and IRJ participated in the MrOS study design and data collection. IRJ and SE participated in the reading of x-rays. SE analyzed the data and wrote the manuscript under the supervision of LJ. SE, IRJ, MK, DM, CO, CT, LEK and LJ interpreted the data and critically reviewed the paper. All authors read and approved the final manuscript.

## Pre-publication history

The pre-publication history for this paper can be accessed here:

http://www.biomedcentral.com/1471-2474/14/352/prepub
